# FireMM-IR: An Infrared-Enhanced Multi-Modal Large Language Model for Comprehensive Scene Understanding in Remote Sensing Forest Fire Monitoring

**DOI:** 10.3390/s26020390

**Published:** 2026-01-07

**Authors:** Jinghao Cao, Xiajun Liu, Rui Xue

**Affiliations:** 1 School of Mechanical Engineering, Jiangsu University of Science and Technology, Zhenjiang 212000, China; 2 School of Electronic Science and Engineering, Nanjing University, Nanjing 210023, China; 3 School of Internet of Things Engineering, Jiangnan University, Wuxi 214122, China; 4 School of Energy and Power Engineering, Nanjing Institute of Technology, Nanjing 211167, China

**Keywords:** multi-modal large language model, forest fire understanding, infrared-aided

## Abstract

**Highlights:**

**What are the main findings?**

- First remote sensing multi-modal large language model for forest fire scene understanding: FireMM-IR is the first MLLM specifically designed for remote sensing forest fire imagery, enabling high-level scene understanding that integrates descriptive, analytical, and predictive insights—capabilities that conventional deep learning-based RS fire detection or segmentation models cannot provide.
- Methodological superiority in RS applications: By fusing infrared and optical remote sensing data through a dual-modality encoder, incorporating a Class-Aware Memory (CAM) module for context-aware reasoning, and a multi-task instruction head for description, prediction, and pixel-level segmentation, FireMM-IR achieves state-of-the-art performance in remote sensing fire monitoring.

**What are the implications of the main findings?**

- Advances RS fire monitoring from perception to holistic scene understanding: FireMM-IR overcomes the limitations of conventional RS deep learning models, offering interpretable, instruction-driven insights into fire evolution, intensity, and spatial distribution.
- Provides actionable remote sensing solutions for disaster management: The combination of multi-spectral fusion, contextual memory, and multi-task learning allows accurate fire localization, predictive reasoning, and high-precision analysis, supporting timely decision-making in real-world RS-based forest fire scenarios.

**Abstract:**

Forest fire monitoring in remote sensing imagery has long relied on traditional perception models that primarily focus on detection or segmentation. However, such approaches fall short in understanding complex fire dynamics, including contextual reasoning, fire evolution description, and cross-modal interpretation. With the rise of multi-modal large language models (MLLMs), it becomes possible to move beyond low-level perception toward holistic scene understanding that jointly reasons about semantics, spatial distribution, and descriptive language. To address this gap, we introduce FireMM-IR, a multi-modal large language model tailored for pixel-level scene understanding in remote-sensing forest-fire imagery. FireMM-IR incorporates an infrared-enhanced classification module that fuses infrared and visual modalities, enabling the model to capture fire intensity and hidden ignition areas under dense smoke. Furthermore, we design a mask-generation module guided by language-conditioned segmentation tokens to produce accurate instance masks from natural-language queries. To effectively learn multi-scale fire features, a class-aware memory mechanism is introduced to maintain contextual consistency across diverse fire scenes. We also construct FireMM-Instruct, a unified corpus of 83,000 geometrically aligned RGB–IR pairs with instruction-aligned descriptions, bounding boxes, and pixel-level annotations. Extensive experiments show that FireMM-IR achieves superior performance on pixel-level segmentation and strong results on instruction-driven captioning and reasoning, while maintaining competitive performance on image-level benchmarks. These results indicate that infrared–optical fusion and instruction-aligned learning are key to physically grounded understanding of wildfire scenes.

## 1. Introduction

Multi-modal large language models (MLLMs) have recently achieved impressive progress in visual understanding, showing strong capabilities in multi-modal reasoning tasks such as image captioning, visual question answering (VQA), and visual grounding [1]. By leveraging large-scale pre-training on paired vision–language data, these models can align textual and visual semantics and perform diverse tasks through natural language instructions. In the field of remote sensing (RS), MLLMs such as RSGPT [2], GeoChat [3], SkyEyeGPT [4], and EarthGPT [5] have extended these capabilities to satellite imagery, enabling image-level and region-level understanding. Despite their success, most existing RS MLLMs remain limited to static perception tasks such as captioning or region grounding, lacking the ability to perform high-level reasoning and fine-grained scene analysis that are essential in dynamic environmental applications such as forest fire monitoring.

Forest fire monitoring represents a critical yet challenging task in RS image understanding [6]. Fires are highly dynamic, spatially heterogeneous, and influenced by complex environmental conditions such as terrain, vegetation, wind, and smoke. Early detection and accurate understanding of fire evolution are essential for disaster management and ecological protection [7,8,9]. Traditional fire monitoring methods based on spectral indices (e.g., NBR [10], NDVI [11]) or handcrafted infrared thresholds can detect hotspots but fail to interpret fire behavior or evolution. With the rise of deep learning, CNN- and Transformer-based models have achieved notable improvements in fire detection and burned area mapping. However, these task-specific perception models still exhibit several limitations: (1) they rely on fixed task formulations (e.g., binary classification or semantic segmentation) and cannot generalize to unseen fire scenarios; (2) they lack interpretability and contextual reasoning ability to analyze fire causes, spread directions, or intensity variations; and (3) they cannot integrate heterogeneous modalities such as infrared and optical data in a unified reasoning framework. Furthermore, infrared imaging also has important physical limitations. Fire-generated smoke can partially attenuate or scatter the thermal signal, especially in dense plumes, and near-sensor saturation may occur in extremely high-temperature cores or when the dynamic range is limited. In addition, a single thermal snapshot only captures the current heat distribution and cannot directly reveal the temporal evolution of the fire front. In practice, effective propagation prediction requires multi-temporal observations to track the spatio-temporal progression of hotspots under changing wind, fuel, and atmospheric conditions. These considerations further motivate the use of multi-modal RGB–IR information and, in future work, the integration of truly temporal models.

Consequently, there is an urgent need to transition from traditional deep learning–based perception models to multi-modal large models capable of advanced scene understanding, where the system can describe, analyze, and predict fire situations through interactive natural language reasoning.

To this end, we propose FireMM-IR, an infrared-enhanced multi-modal large language model designed for comprehensive scene understanding and prediction in remote sensing forest fire scenarios. FireMM-IR integrates a dual-modality encoder that fuses optical and infrared data, enabling the model to capture both visible and latent fire features under smoke or nighttime conditions. On top of this encoder, we design a multi-task instruction head that jointly supports (1) fire scene description, generating coherent textual summaries of the fire situation; (2) fire analysis and prediction, providing reasoning-based insights into fire intensity, spatial evolution, and potential spread trends; and (3) pixel-level segmentation, accurately localizing fire regions and their spatial extent under natural language guidance. This unified framework allows FireMM-IR to serve as an intelligent dialogue system for RS-based fire monitoring, capable of producing both descriptive and spatial outputs from user queries such as “Describe the current fire situation,” “Predict the possible spread direction,” or “Segment the burning area.”

To enhance spatial and semantic consistency across different fire scales, FireMM-IR employs a Class-Aware Memory (CAM) mechanism, which learns and stores contextual geo-representations of fire patterns across the training dataset. This mechanism enables the model to retrieve high-level contextual cues during inference, thereby improving segmentation coherence and analytical accuracy in complex fire scenes.

A key challenge in developing such models lies in the scarcity of large-scale RS datasets that simultaneously include multi-spectral imagery, textual annotations, and pixel-level labels. To address this, we construct the FireMM-Instruct dataset, a comprehensive multi-spectral forest fire dataset comprising 83,000 image pairs and corresponding annotated instances. Each instance is paired with detailed fire descriptions, bounding boxes, and pixel-level segmentation masks, enabling instruction-tuned multi-task learning for descriptive, analytical, and spatial reasoning tasks. To ensure linguistic diversity and factual accuracy, we adopt a description generation pipeline based on GPT-4o to automatically produce fire-related textual annotations, followed by manual verification and fine-tuning to improve semantic alignment and consistency.

In optimizing multi-task pixel-level MLLMs, an inherent training imbalance problem exists: text generation converges rapidly, whereas segmentation requires extended training for spatial precision. To resolve this, FireMM-IR introduces a two-stage training strategy. In Stage I, the model is trained primarily for text generation and reasoning to acquire domain-level multi-modal alignment, while light segmentation supervision provides initial mask learning. In Stage II, the focus shifts toward improving spatial segmentation accuracy and predictive reasoning by increasing the ratio of pixel-level data and training iterations. This staged optimization effectively balances linguistic and spatial learning objectives, leading to robust multi-task convergence.

**Figure 1:**
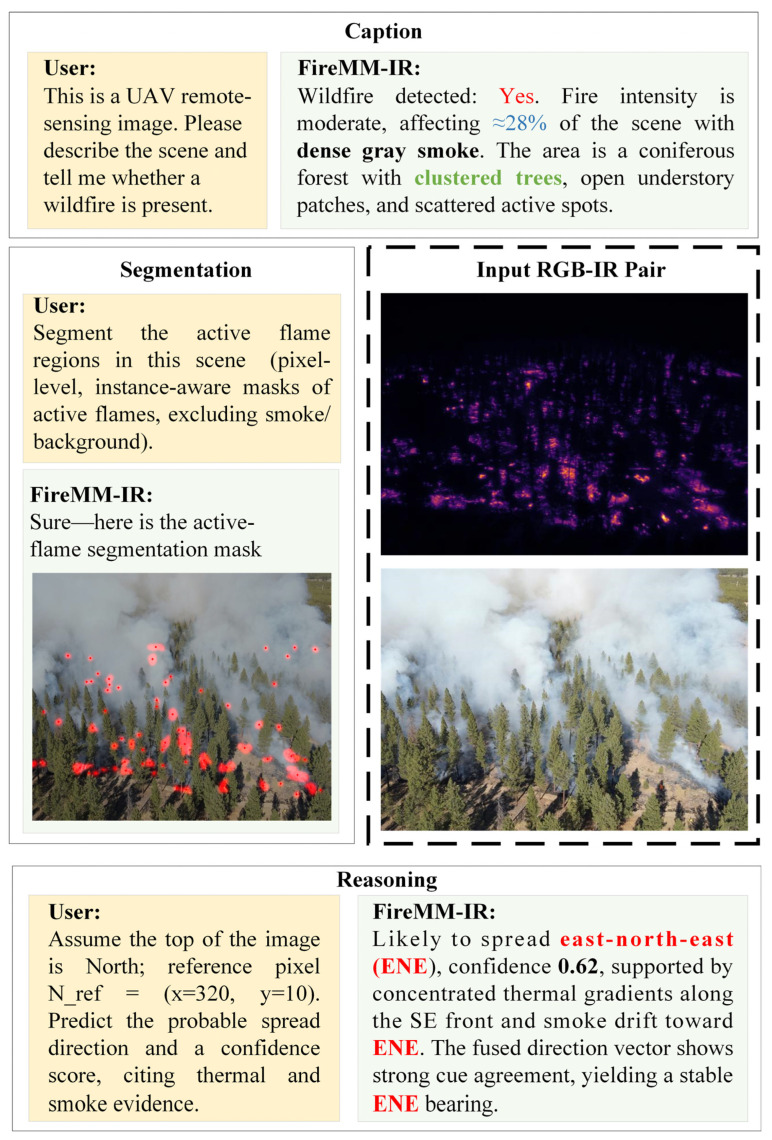
FireMM-IR task overview on a paired UAV RGB–IR scene. Given natural instructions, FireMM-IR produces (i) a caption indicating wildfire presence, intensity, coverage, and smoke, (ii) pixel-level active-flame masks, and (iii) a spread-direction reasoning with confidence from fused infrared/smoke cues.

To visually ground the proposed tasks, Figure 1 illustrates FireMM-IR on a paired UAV RGB–IR scene. Given a natural instruction, the model (i) produces an instruction-aligned caption indicating wildfire presence, intensity, coverage, and smoke status; (ii) outputs pixel-accurate, instance-level masks for active flames; and (iii) performs spread-direction reasoning with confidence by fusing infrared and smoke cues. This tri-task example highlights FireMM-IR’s shift from static perception to holistic, physically grounded understanding.

Extensive experiments show that FireMM-IR achieves state-of-the-art performance across multiple remote sensing benchmarks, particularly excelling in descriptive captioning, analytical fire prediction, and precise pixel-level segmentation. Moreover, it maintains competitive image-level understanding capabilities compared to leading RS MLLMs. By integrating infrared perception, contextual memory reasoning, and instruction-driven multi-task learning, FireMM-IR establishes a new paradigm for intelligent remote sensing fire monitoring—transforming fire recognition from low-level detection to holistic understanding and predictive analysis.

### Contributions

The main contributions of this work are summarized as follows:

- We propose FireMM-IR, an infrared-enhanced multi-modal large language model for remote sensing forest fire monitoring, capable of unified scene understanding that integrates fire description, fire analysis and prediction, and pixel-level localization.
- We design a Class-Aware Memory (CAM) module that captures class-wise contextual information and enhances the semantic and spatial consistency of multi-scale fire understanding.
- We construct FireMM-Instruct, a large-scale multi-spectral dataset providing paired textual descriptions, bounding boxes, and pixel-level masks for forest fire scenes, enabling comprehensive instruction-tuned learning.
- We develop a two-stage training strategy to balance text-based reasoning and pixel-level segmentation objectives, achieving superior performance in both descriptive and analytical fire monitoring tasks.

In this work, FireMM-IR performs static inference on individual RGB–IR frames that capture instantaneous fire conditions. Although the underlying FLAME sequences provide multi-temporal coverage, we do not explicitly model temporal dynamics (e.g., recurrent or sequential prediction) in the current architecture. Instead, our focus is on accurate per-frame segmentation, captioning, and reasoning, which can be used as building blocks for future multi-temporal propagation models.

## 2. Related Works

### 2.1. Forest-Fire Perception in Remote Sensing

Forest fire monitoring with remote sensing has a long history rooted in spectral-index analysis and infrared-anomaly detection. Classical methods use indices such as the Normalized Burn Ratio (NBR) [10] and vegetation indices (e.g., NDVI) [11] to delineate burned areas and assess burn severity, while infrared thresholds from instruments like MODIS/VIIRS [12,13] are widely employed for active fire detection and near-real-time reporting (e.g., NASA [14] FIRMS [15]). These rule-based and physics-inspired algorithms provide robust, operational fire products at large scales but are limited in complex conditions such as heavy smoke, mixed pixels, or nighttime observations where purely spectral rules may miss latent hotspots or generate false alarms.

The advent of deep learning has substantially improved fire detection and mapping in remote sensing. Convolutional neural networks (CNNs) [16] and vision transformers [17] have been applied to classification [18], object detection (smoke/fire spots) [19], and semantic/instance segmentation [20] of burned or burning areas, often outperforming handcrafted-feature methods in benchmark tasks. Recent surveys summarize that deep models can increase sensitivity and spatial detail, especially when trained on high-resolution airborne or UAV imagery [21]. However, these deep learning approaches are typically task-specific (e.g., detection or segmentation) and rely on supervised training with large annotated datasets; they frequently struggle to generalize to unseen fire scenarios, to provide interpretable reasoning about fire behavior, or to fuse heterogeneous modalities effectively at the reasoning level. Additionally, false positives due to scene confounders (e.g., sun glint, hot bare soil) and the scarcity/diversity of annotated multi-spectral datasets remain major hurdles.

Because fires manifest differently across spectral bands and scales, multisensor and multi-spectral fusion—particularly combining infrared with optical bands—has been shown to enhance both early detection and characterization of fire intensity and pre-fire anomalies. Studies demonstrate that fusing infrared radiometry (which directly senses heat) with higher-resolution multispectral imagery yields improved localization of hotspots, better discrimination of smoke-obscured ignition points, and more accurate estimation of fire-related surface properties [22]. Despite these gains, most fusion methods focus on improving per-pixel detection or mapping accuracy rather than enabling higher-level semantic interpretation or predictive reasoning about fire evolution.

High-quality datasets are a prerequisite for training robust models. Operational satellite products such as MODIS/VIIRS [12,13] active-fire catalogs and services like FIRMS supply continuous global alerts useful for large-scale monitoring, but their spatial resolution and product semantics (point/infrared alerts) limit their utility for pixel-precise segmentation and instance-level analysis. To address this, recent research efforts have produced higher-resolution and task-specific datasets for wildfire segmentation and RGB–infrared benchmarks (e.g., Land8Fire [23] and UAVs-FFDB [24]), which enable supervised training of segmentation and fusion models; nevertheless, large-scale multi-spectral datasets that also include instance-level textual descriptions and instruction-aligned labels remain scarce.

Taken together, prior work shows three clear gaps that motivate moving beyond conventional deep models toward instruction-capable, multi-modal understanding systems for RS fire monitoring: (1) classical and deep-learning approaches are largely perception-centric and task-specialized, (2) although multispectral fusion improves detection, it is rarely integrated into frameworks that support high-level semantic reasoning or prediction, and (3) there is a shortage of large-scale, multi-modal, instance-annotated datasets that enable language-conditioned, pixel-level supervision. These gaps limit the ability of existing RS systems to provide interpretable, multi-modal descriptions and predictive analyses required for decision-making in complex fire scenarios. The FireMM-IR work addresses these gaps by combining infrared–optical fusion, a context-aware memory for geo-semantic consistency, and an instruction-driven multi-task head trained on a purpose-built multi-spectral dataset to enable descriptive, analytical, and pixel-precise fire understanding.

### 2.2. Remote Sensing Multi-Modal Large Language Models

In recent years, multi-modal large language models (MLLMs) have achieved significant progress in integrating vision and language for remote sensing (RS) applications. These models leverage pre-trained language-vision architectures to perform a wide range of tasks, including image captioning, visual question answering, and visual grounding. Early attempts, such as RSGPT, adapted pre-trained MLLMs to the RS domain, supporting image-level captioning and question answering. Subsequent models, including GeoChat [2], SkyEyeGPT [3], EarthGPT [4], and Lhrs-Bot [24,25,26], extended these capabilities to the region-level, enabling region-specific visual question answering, grounded image captioning, and bounding-box localization of instances according to user instructions. These developments demonstrate the potential of MLLMs to provide interactive multi-modal understanding in RS, moving beyond conventional perception methods that are limited to single-task outputs.

Despite these advances, existing RS MLLMs remain constrained in several key aspects. First, most models are limited to image- or region-level tasks and lack pixel-level dialogue or segmentation capabilities. While some recent work, such as GeoPix [27], has explored pixel-level interaction through referring segmentation, these efforts typically handle single-instance outputs and do not provide comprehensive multi-task reasoning. Second, the majority of RS MLLMs primarily focus on optical imagery, and few leverage multi-sensor modalities such as infrared, which are crucial for environmental monitoring tasks like forest fire detection and analysis. Third, large-scale multi-modal RS datasets suitable for instruction-driven learning are scarce; most available datasets provide only image-level labels or bounding boxes, lacking the joint text, spatial, and pixel-level annotations necessary for unified multi-task training.

To address these limitations, we propose FireMM-IR, a remote sensing MLLM specifically designed for forest fire scenarios. Unlike existing models, FireMM-IR integrates infrared and optical data to capture both visible and obscured fire regions, enabling more accurate perception under challenging conditions such as smoke or nighttime observations. The model further incorporates a Class-Aware Memory (CAM) module, which maintains geo-semantic context across multi-scale fire instances, ensuring spatial and semantic consistency in both segmentation and analytical tasks. In addition, FireMM-IR is trained on FireMM-Instruct, a purpose-built multi-spectral dataset that provides joint annotations of textual descriptions, bounding boxes, and pixel-level masks, supporting instruction-driven multi-task learning. Finally, a two-stage training strategy balances the optimization of text generation and pixel-level segmentation, overcoming the conflict commonly observed when training MLLMs for combined language and vision tasks.

By addressing these critical gaps, FireMM-IR extends the capabilities of RS MLLMs from image- and region-level understanding to holistic scene comprehension, encompassing fire description, analysis, prediction, and pixel-level localization. Compared to existing models, it offers a unique combination of multi-sensor fusion, memory-enhanced context reasoning, and instruction-aligned multi-task learning, establishing a new paradigm for remote sensing forest fire monitoring.

## 3. Methodology

### 3.1. Overall Architecture

The overall architecture of FireMM-IR is designed as an infrared-enhanced multi-modal large language model (MLLM) for comprehensive scene understanding and prediction in remote sensing forest fire monitoring.

**Figure 2:**
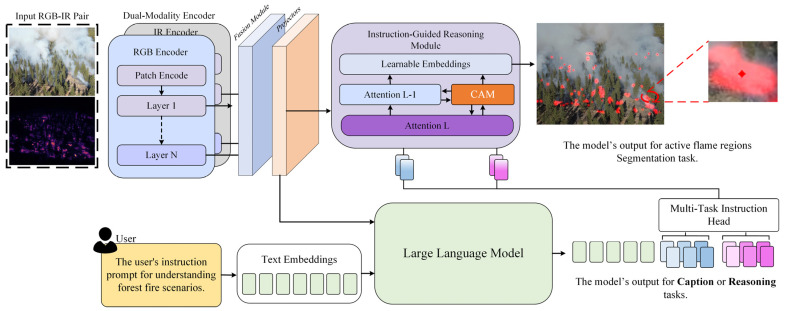
Overall architecture of FireMM-IR. The model processes a paired RGB–IR input through a dual-modality encoder, which extracts and fuses optical and infrared features. The Instruction-Guided Reasoning Module performs cross-attention between instruction tokens and fused features, while the Class-Aware Memory (CAM) helps retain context-specific patterns. The model outputs tasks such as active flame segmentation, captioning, and reasoning through the Multi-Task Instruction Head. The red region indicates the fire location predicted by the model, while the arrows and symbols represent the direction of data flow.

Figure 2 illustrates the overall architecture of FireMM-IR and the end-to-end workflow from input data to outputs. Given a paired RGB–IR frame that has been temporally aligned and geometrically co-registered in the preprocessing stage, FireMM-IR first feeds the optical RGB image and the infrared (IR) temperature map into a dual-modality encoder to extract modality-specific feature maps. A temperature-aware fusion module then combines these features into a unified multi-modal representation. On top of this representation, the Instruction-Guided Reasoning Module performs cross-attention between the instruction tokens and the fused visual features, while the Class-Aware Memory (CAM) injects global context via a set of learnable prototypes. Finally, a Multi-Task Instruction Head decodes the shared representation into three outputs: pixel-wise active flame segmentation, detailed scene captioning, and high-level reasoning (including fire-spread direction). Given an RGB–infrared image pair $\left\{I_{rgb},I_{IR}\right\}$ and an instruction, $\;T_{init}$, FireMM-IR produces three complementary outputs—caption $Y_{cap}$, reasoning $Y_{reason}$, and segmentation mask $M_{seg}$:

(1) $${\{I}_{rgb},I_{IR},T_{init}\}\overset{E_{dual}}{\to }F_{fused}\overset{LLM+CAM}{\to }\{Y_{cap},Y_{reason},M_{seg}\}$$

In line with recent multimodal LLM works, we use the term “reasoning” to describe the third output branch, which combines a discrete prediction with an explicit natural-language explanation. In our wildfire setting, this branch essentially performs spread-direction inference: given a single RGB–IR frame, it predicts a discretized spread-direction label in the image plane and generates a short textual rationale that is grounded in the visible and thermal evidence. It does not attempt to model full physical fire propagation over time, and should therefore be interpreted as an explanation-augmented prediction head rather than a complete propagation simulator.

The Dual-Modality Encoder $E_{dual}$ first extracts spectral–spatial representations from both optical and infrared domains. The optical encoder captures fine-grained spatial and textural features, while the infrared encoder focuses on infrared emissions that reveal latent fire sources under smoke or nighttime conditions.

Their outputs are integrated via a fusion module to form a unified embedding space $F_{fused}\in R^{H\times W\times D}$, ensuring that visible and infrared cues jointly contribute to subsequent reasoning. Next, the Multi-Task Instruction Head maps $F_{fused}$ into the latent space of the large language model (LLM). Through cross-attention between instruction tokens and fused features, the model dynamically adapts to user prompts such as “Describe the fire scene,” “Predict the spread direction,” or “Segment the burning area.”

This mechanism enables the LLM backbone (LLaVA-based) to perform contextual reasoning conditioned on both textual input and multi-spectral evidence. To enhance semantic and spatial coherence, the Class-Aware Memory (CAM) module operates atop the fused representation.

CAM maintains a memory bank of class-specific contextual embeddings that encode representative patterns of different fire states (e.g., low, medium, high intensity, and smoke-dominant). During inference, CAM retrieves and integrates these embeddings through attention-weighted fusion, reinforcing spatial consistency and improving reasoning robustness across diverse fire scales. Finally, the Multi-Task Instruction Head decodes three outputs under unified supervision:

(1) A caption describing the current fire situation;
(2) A reasoning statement predicting intensity evolution and potential spread;
(3) A segmentation mask localizing active fire regions.

All outputs are jointly optimized with a composite objective that balances linguistic generation and spatial accuracy.

In summary, FireMM-IR forms a cohesive architecture that bridges semantic understanding and pixel-level perception in remote sensing forest fire imagery. Through infrared-enhanced representation learning, instruction-guided fusion, and memory-based contextual reasoning, the framework enables interpretable and predictive fire monitoring beyond conventional detection pipelines.

### 3.2. Dual-Modality Encoder

The Dual-Modality Encoder in FireMM-IR is designed to jointly extract and fuse optical and infrared information, enabling consistent scene understanding across diverse illumination and atmospheric conditions.

Unlike single-modality encoders in conventional remote sensing MLLMs, FireMM-IR employs a dual-stream architecture that integrates RGB texture cues with infrared radiometric patterns, thus capturing both visible and latent fire dynamics. Given an RGB image and its aligned infrared temperature map, the two modality-specific encoders extract feature maps.

Given an RGB image and its aligned infrared temperature map, two independent Vision Transformers (ViT-B/16 backbones) are used to extract modality-specific features:

(2) $$F_{rgb}=E_{rgb}(I_{rgb}),\;\;F_{IR}=E_{IR}(I_{IR})\;$$

After independent encoding, modality features are fused through an adaptive weighting scheme:

(3) $$F_{fuse}=\alpha F_{rgb}+(1-\alpha )Proj(F_{IR})$$

where α ∈ [0, 1] is a learnable scalar controlling modality dominance and Proj(·)  aligns the channel dimension of $F_{IR}$ to match $F_{rgb}\;$. This adaptive fusion dynamically emphasizes RGB information in clear conditions and infrared information under smoke or low visibility. To further refine alignment between the two modalities, an attention-based Cross-Modality Spatial Alignment (CMSA) layer is introduced.

For each spatial location, infrared features are warped toward the RGB reference via:

(4) $$F_{IR}^{aligned}=Softmax(\frac{Q_{rgb}K_{IR}^{T}}{\sqrt{d}})V_{IR}$$

where $Q_{rgb}$, $K_{IR}^{T}$, and $V_{IR}$ are the query, key, and value projections of the respective modality features. This process corrects sub-pixel offsets caused by sensor differences and enhances the accuracy of fire boundary localization.

The resulting fused representation $F_{fused}$ is flattened into a sequence of tokens $F={\left\{f_{i}\right\}}_{i=1}^{N}$, where N = H′ × W′, and projected into the instruction-conditioned embedding space through a lightweight adapter. These tokens are then passed to the Multi-Task Instruction Head, enabling cross-modal reasoning, descriptive captioning, and pixel-level segmentation.

Given an aligned RGB image and its corresponding IR temperature map, we use two backbone encoders to extract modality-specific feature maps. To exploit the physical meaning of the IR signal, we design a temperature-aware gating mechanism that predicts a spatial gating mask from the IR features. This gating mask highlights high-temperature regions that are likely to correspond to active combustion or residual heating, while suppressing cooler background regions that may still appear visually bright (e.g., sunlit vegetation or reflective surfaces). We then use this IR-derived mask to modulate the RGB feature maps before cross-modal fusion, effectively steering the fused representation towards physically meaningful hotspots. In this way, the infrared channel does not simply act as an additional input, but actively guides the fusion process based on local temperature patterns.

### 3.3. Instruction-Guided Reasoning and Contextual Memory Mechanism

**Figure 3:**
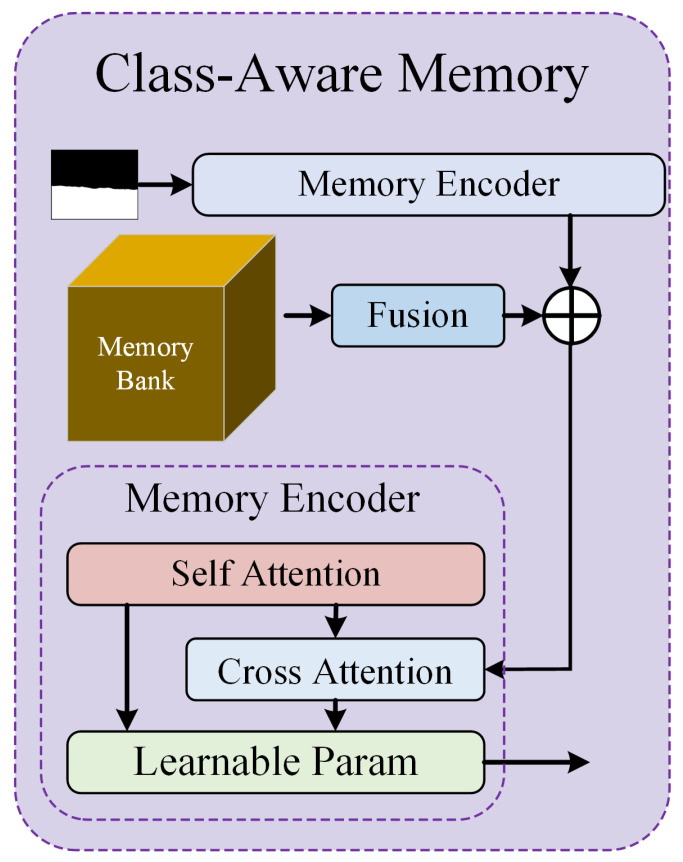
The architecture of CAM proposed in this paper.

To achieve unified scene understanding and predictive analysis in forest fire monitoring, FireMM-IR integrates an Instruction-Guided Reasoning Module with a Class-Aware Memory (CAM) Mechanism. This combination enables the model to interpret natural language instructions, generate descriptive and analytical outputs, and maintain semantic consistency across fire scenes of varying scales and intensities. As shown in Figure 3, the proposed CAM module maintains a set of class-aware prototypes that summarize typical visual–thermal patterns observed across the training set (e.g., active fire fronts, smoke plumes, different vegetation/background types). During inference, each pixel-level fused feature performs cross-attention to this prototype memory, producing a refined representation that is anchored to these global semantic concepts. This mechanism encourages pixels with similar visual–thermal patterns to attend to similar prototypes, which promotes consistent semantic assignments across the scene. In practice, CAM leads to smoother and more coherent segmentation masks (e.g., fewer isolated false positives and more continuous fire boundaries) and also stabilizes the features that are fed into the LLM head, thereby improving coherence between the predicted masks and the generated textual descriptions. The Instruction-Guided Reasoning Module serves as the interface between the visual encoder and the language model. Given the fused multi-spectral features and instruction tokens, a cross-attention mechanism aligns them in the shared embedding space:

(5) $$h_{v\to t}=A(Q_{t},\;K_{v},V_{v})$$

where A(⋅) denotes the multi-head attention operation. This alignment ensures that each linguistic query dynamically attends to its corresponding visual evidence—whether describing active flames, reasoning about potential spread, or localizing regions for segmentation. A lightweight adapter projects the fused representation into the embedding space of the LLaVA-1.5, enabling multi-task outputs without retraining the base LLM. Through this design, FireMM-IR supports natural language-driven tasks including captioning (“Describe the current fire intensity”), reasoning (“Predict the possible spread direction”), and segmentation (“Locate active fire regions”).

To enhance contextual coherence and improve segmentation consistency, the Class-Aware Memory (CAM) introduces a learnable memory bank $M={\left\{m_{c}\right\}}_{c=1}^{C}$, where each prototype mcm_cmc encodes the global semantics of a specific fire-related class (e.g., *flame*, *smoke*, *background*). During training, local feature embeddings fif_ifi are refined by retrieving relevant memory prototypes through similarity-based fusion:

(6) $${\hat{f}}_{i}=f_{i}+\lambda \sum_{c=1}^{C}w_{ic}m_{c},\;\;\;\;where\;w_{ic}=\frac{exp(f_{i}^{T}m_{c}/\tau )}{\sum_{k}exp(f_{i}^{T}m_{k}/\tau )}$$

Here, λ controls the fusion strength and τ regulates attention sharpness. The temperature parameter τ controls the sharpness of the attention/gating distribution; in all experiments we fix τ to a constant value selected on the validation set to balance stability and selectivity.

This memory-guided refinement introduces dataset-level contextual priors, stabilizing predictions across different fire scales and atmospheric conditions. It allows the model to recognize small ignition sources, preserve flame continuity, and differentiate active fire from smoke-obscured regions. For pixel-level understanding, the reasoning module outputs a set of segmentation tokens, each corresponding to an instance described in the instruction. The attention map associated with each token is decoded into a binary mask:

(7) $$\;{\tilde{M}}_{k}=\sigma (W_{m},A_{k})$$

where $A_{k}$ is the attention activation map for token k and $W_{m}$ is a linear projection layer. By conditioning mask generation on both language semantics and memory-augmented visual features, FireMM-IR achieves precise fire localization and consistent boundary delineation across diverse scenes.

### 3.4. Multi-Task Instruction Head

The Multi-Task Instruction Head serves as the integrative output layer of FireMM-IR, enabling the model to flexibly execute captioning, reasoning, and pixel-level segmentation within a unified instruction-driven framework.

It interprets multimodal embeddings from the reasoning module and dynamically adapts to task-specific outputs, thus bridging descriptive and spatial understanding under natural language control.

After fusion by the Instruction-Guided Reasoning Module, the joint representation $H=\{h_{i}\}$ contains both spectral–spatial cues and encoded instruction semantics. To determine which type of output should be generated, an instruction classifier first estimates the task probability distribution:

(8) $$p(task|T)=Softmax(W_{t}\cdot MeanPool(T)+b_{t})$$

where $W_{t}$ and $b_{t}$ are learnable parameters and T is the instruction token sequence. Based on this routing, the head selectively activates one of three branches for captioning, reasoning, or segmentation.

For captioning and reasoning, the model conditions the large language model (LLM) on the visual embeddings and generates text autoregressively. Given the context C={H,T}, the generative process follows:

(9) $$P(Y|C)=\prod_{i=1}^{L}P(y_{i}|y<i,C)$$

where $Y={\left\{y_{i}\right\}}_{i=1}^{L}$ denotes the output token sequence.

This branch enables FireMM-IR to produce descriptive fire summaries (“A moderate fire with dense smoke in the northwest region”) and analytical reasoning (“The fire is likely to expand toward the slope due to uphill wind flow”).

Fine-tuning with instruction–caption pairs from FireMM-Instruct ensures that textual outputs are both contextually grounded and physically consistent.

For pixel-level segmentation, the head refines the intermediate masks ${\tilde{M}}_{k}$ (introduced in Section 3.3) into final trainable predictions $M_{k}$.

Each segmentation token $s_{k}$ attends to spatial features to produce an activation map $A_{k}$, which is decoded into a binary mask as:

(10) $$M_{k}^{\left(out\right)}=\sigma (W_{m}A_{k})$$

where $W_{m}$ is a learnable projection matrix and σ(·) is the sigmoid activation.

Each mask token is semantically grounded in the textual query, enabling instruction-based spatial localization such as “highlight active burning zones” or “mark smoke diffusion areas.”

The three task branches are optimized jointly using a composite multi-objective loss:

(11) $$L_{total}=\lambda_{1}L_{cap}+\lambda_{2}L_{reason}+\lambda_{3}L_{seg}$$

where $L_{cap}$ is the language modeling loss for captioning, $L_{reason}$ the consistency loss for analytical inference, and $L_{seg}$ the binary cross-entropy loss for segmentation. The weighting coefficients $\lambda_{i}$ are dynamically adjusted according to training stage to maintain convergence balance between linguistic and spatial learning objectives. Here, λ_1_, λ_2_, and λ_3_ control the relative weights of the captioning, directional inference, and segmentation losses in the general multi-task objective of Equations (11)–(13) then instantiate different settings of these weights for the first and second training phases, respectively, so the λ’s are related but not identical across the two stages.

In essence, the Multi-Task Instruction Head acts as the unified output interface of FireMM-IR. It translates multimodal representations into diverse yet coherent outputs—textual, analytical, and spatial—depending on user instruction. By jointly optimizing these tasks, FireMM-IR attains flexible scene comprehension and predictive reasoning, advancing remote sensing fire monitoring toward holistic multi-modal intelligence.

### 3.5. Two-Stage Optimization Strategy

To effectively combine the RGB–IR encoders with the large language model, we adopt a two-phase training strategy. In the first phase, we focus on stabilizing the dual-modality visual backbone and the segmentation head using dense pixel-wise supervision on FireMM-Instruct. This phase learns reliable multi-modal representations and accurate active flame masks. In the second phase, we adapt the LLM head to the wildfire domain by instruction tuning on the captioning and reasoning tasks, while keeping the low-level encoders fixed or only lightly fine-tuned. This design prevents catastrophic forgetting of visual features, and allows the LLM to specialize in fire-specific descriptions (e.g., fire intensity, affected area, smoke conditions) and directional reasoning, using the CAM-enhanced fused representation as its visual context.

With the multi-task loss function $L_{total}$ defined in Section 3.4, the training process of FireMM-IR focuses on balancing two inherently different objectives—language-based reasoning and pixel-level segmentation. Since text generation tasks usually converge faster while spatial segmentation requires prolonged optimization for fine-grained precision, a uniform training schedule can easily lead to overfitting on textual tasks or underfitting on spatial ones. To resolve this imbalance, FireMM-IR adopts a two-stage optimization strategy that progressively transitions from semantic-level alignment to spatial-level refinement, ensuring stable convergence and consistent multimodal understanding.

In Stage I (Text & Reasoning Pre-Alignment), the model concentrates on building strong vision–language correspondence. The captioning and reasoning heads dominate the optimization process, enabling the model to learn how spectral–spatial cues from the RGB–IR encoder relate to descriptive and analytical text representations. A lightweight segmentation branch is also included at this stage but with a very small loss weight to provide weak spatial supervision without distracting the language alignment process. The overall loss can be expressed as

(12) $$L_{stage1}=\lambda_{1}L_{cap}+\lambda_{2}L_{reason}+\epsilon L_{seg}$$

where ϵ≪1 introduces minimal pixel-level guidance. Through this stage, FireMM-IR learns to generate accurate and context-aware fire scene descriptions and analytical reasoning sentences while establishing a stable multimodal embedding space that aligns spectral features with textual semantics.

After the language and visual modalities are well aligned, the training proceeds to Stage II (Pixel-Level Refinement), which focuses on enhancing segmentation precision and spatial coherence. In this stage, the segmentation loss receives a significantly higher weight, and the proportion of pixel-labeled samples in each batch is increased. The updated optimization objective becomes

(13) $$L_{stage2}=\lambda_{1}^{\prime }L_{cap}+\lambda_{2}^{\prime }L_{reason}+\lambda_{3}^{\prime }L_{seg},\;where{\;\lambda }_{3}^{\prime }\gg \lambda_{1}^{\prime },\lambda_{2}^{\prime }\;$$

A hybrid loss formulation combining binary cross-entropy and Dice loss is used to further improve mask boundary fidelity:

(14) $$L_{seg}=L_{BCE}+\gamma L_{Dice}$$

During this phase, a lower learning rate is applied to the encoders to preserve the previously learned multimodal alignment, while the segmentation layers and the Class-Aware Memory (CAM) module are fine-tuned to enhance local structure understanding and region consistency.

Overall, this two-stage optimization scheme allows FireMM-IR to first master global semantic reasoning and then progressively refine its spatial prediction capability. The result is a well-balanced model that produces coherent textual analyses, accurate fire reasoning, and geometrically consistent segmentation masks—achieving unified understanding and prediction in remote sensing forest fire scenarios.

## 4. Dataset

### 4.1. Motivation and Requirements

Forest fires are complex and rapidly evolving phenomena that can span from small ignition points to large-scale conflagrations across diverse terrains. In remote sensing imagery, these events present significant challenges due to their multi-scale spatial patterns, dynamic evolution, and environmental obscurants such as smoke, clouds, and atmospheric haze. Effective monitoring in this domain requires multi-sensor data, where optical imagery captures high-resolution visual cues of the landscape and flames, while infrared imagery reveals latent heat sources and hidden ignition points that may be obscured in the visible spectrum or occur at night. Although satellite and airborne remote sensing data are increasingly available, existing datasets are largely limited to single-task annotations such as burned area masks or bounding boxes, and they rarely provide instruction-aligned, multi-task labels or instance-level masks that enable integrated reasoning, descriptive captioning, and precise localization—capabilities essential for building remote sensing multi-modal large language models (RS-MLLMs) capable of holistic scene understanding.

To address these limitations, the FireMM-Instruct dataset is designed with three primary objectives tailored to remote sensing forest fire monitoring. First, it supports instruction-driven multi-task learning, enabling models to perform fire description (captioning), fire analysis and prediction (reasoning), and fire localization (pixel-level segmentation) within a unified framework. Each scene in the dataset is associated with textual descriptions summarizing fire intensity, spatial distribution, and potential spread, while simultaneously providing instance-level masks and bounding boxes for active fire regions. Second, the dataset captures multi-scale spatial distributions and multi-sensor modalities, ensuring that models can reason across small ignition points and large fire clusters, integrating optical and infrared information for robust perception. Finally, FireMM-Instruct is large-scale, diverse, and quality-controlled, encompassing various vegetation types, terrain conditions, temporal variations, and fire intensities. By combining multi-task, multi-modal, and instruction-aligned annotations, the dataset provides a strong foundation for training RS-MLLMs that move beyond conventional perception models toward holistic remote sensing scene understanding, fire analysis, and predictive reasoning.

### 4.2. Dataset Construction

#### 4.2.1. Data Integration: Unifying FLAME Series into FireMM-Instruct

To construct a large-scale multi-modal remote sensing dataset capable of supporting captioning, reasoning, and pixel-level segmentation, we integrate the public Remote Sensing fire datasets—FLAME 1, FLAME 2, and FLAME 3—into a unified corpus named FireMM-Instruct. Although all three datasets capture forest fire scenes with RGB–infrared pairs, their data organization, annotation protocols, and radiometric calibrations differ significantly. Therefore, a systematic integration pipeline is developed to align geometry, normalize infrared intensity, and unify semantic labels. Quantitatively, FLAME 1 provides approximately $3\times 10^{4}$ pairs, FLAME 2 contributes $5.3\times 10^{4}$ pairs, and FLAME 3 adds 738 radiometric pairs, resulting in a total of about $8.3\times 10^{4}$ aligned RGB–IR image pairs. Each pair consists of an optical frame $\;I_{rgb}\in R^{H\times W\times 3}$ and an infrared temperature map $I_{IR}\in R^{H\times W}$, merged into a unified 4-channel tensor.

To ensure pixel-wise correspondence, temporal alignment is performed under a frame offset threshold of Δt ≤ 0.05 s, followed by geometric co-registration using homography-based feature matching, achieving an average alignment error below 0.5 m. For FLAME 3, the radiometric TIFF format is converted into absolute temperature fields via:

(15) T(x,y)=α·v(x,y)+β, T ϵR(Kelvin)

FireMM-Instruct preserves the binary segmentation mask design of the original FLAME datasets, which labels each pixel as either fire or background:

(16) $$M(x,y)\in \{0,1\},\;M(x,y)=\left\{\begin{array}{c} 1,if(x,y)\;belongs\;to\;active\;flame\;region\\ 0,\;otherwise \end{array}\right\}$$

This consistent labeling allows the dataset to serve as a reliable foundation for pixel-level fire localization and scene-level reasoning tasks. To maintain semantic and radiometric consistency across sources, FireMM-Instruct applies a unified normalization scheme to both RGB and infrared data. The optical channels are standardized by global z-score normalization, while infrared values are normalized within the physical range $\left[T_{min},T_{max}\right]$ as:

(17) $$T^{\prime }=\frac{T-T_{min}}{T_{max}-T_{min}}$$

where $T_{min}=250\;K$ and $T_{max}=900\;K$ represent the effective radiometric limits across all sensors. This enables consistent fusion across varying flight altitudes and camera configurations.

Although the three FLAME datasets all target forest fire monitoring with RGB–IR imagery, they were collected under different flight configurations, camera models, and pairing protocols. As a result, their raw infrared responses and RGB–IR alignment accuracy are not strictly identical. In constructing FireMM-Instruct, we explicitly take these discrepancies into account. For each source, we first follow its metadata or recommended calibration procedure to convert raw thermal values into physically meaningful temperature fields (for FLAME-3, via the linear mapping described in Equation (15)). We then apply per-source normalization and clip all infrared values to a common physical range before re-normalizing them using a unified scheme. This reduces cross-sensor bias in absolute temperature while preserving the relative contrast between active fire regions and background.

#### 4.2.2. Data Annotation and Instruction Alignment

In FireMM-Instruct, captioning and reasoning are treated as two independent yet complementary tasks. Captioning focuses on visually grounded descriptions of observable fire phenomena, while reasoning targets higher-level analytical interpretations derived from spatial and infrared patterns. Even without auxiliary metadata such as wind speed or temporal sequences, the combined RGB–IR imagery provides sufficient cues for both descriptive and inferential annotation, enabling hierarchical language supervision from perception-level recognition to cognition-level understanding.

To generate linguistically diverse and physically consistent captions for remote sensing forest fire imagery, the FireMM-Instruct dataset adopts a multi-template and AI-enhancement strategy that integrates quantitative estimation from infrared data with rule-based template filling and large language model augmentation. Each caption instance describes the visible fire condition within a single RGB–IR image pair using three key parameters: fire intensity level, fire coverage ratio, and smoke presence.

The fire coverage is derived directly from the binary segmentation mask M(x,y), which indicates active flame regions as M(x,y)=1. The coverage ratio $R_{f}$ is computed as

(18) $$R_{f}=\frac{\sum_{x,y}M(x,y)}{H\times W}$$

where ***H*** and ***W*** denote the image height and width, respectively. This ratio reflects the proportion of the scene affected by burning activity and serves as a critical quantitative cue for caption construction.

The fire intensity level is inferred jointly from the infrared temperature field T(x,y) and the coverage ratio $R_{f}$. An integrated fire index $I_{f}$ is defined as:

(19) $$I_{f}=\frac{1}{N_{f}}\sum_{M(x,y)=1}\frac{T(x,y)-T_{min}}{T_{max}-T_{min}}$$

where $N_{f}$ is the number of active fire pixels. Based on the dataset-wide distribution of $R_{f}$ and $I_{f}$, the fire intensity is categorized into three discrete levels:

(20) $$L_{f}=\left\{\begin{array}{c} “mild”,\;I_{f}<0.35\;or\;\;R_{f}<0.05\;\\ “moderate”,\;0.35\leq I_{f}<0.65\\ “intense”,\;I_{f}>0.65 \end{array}\right\}$$

This discretization ensures a consistent mapping between radiometric heat, spatial extent, and linguistic semantics, allowing the generated captions to accurately describe the physical characteristics of fire scenes.

The presence of flames is determined directly from the ground-truth mask—if $R_{f}>0$, the scene is labeled as containing active fire. Smoke presence is detected by analyzing the saturation–brightness (SV) components of the HSV color space. A region is labeled as containing smoke if

(21) $$S_{mean}<\tau_{s}\;and\;\;\;V_{mean}>\tau_{v}$$

where typical thresholds are $\tau_{s}$ = 0.25 and $\tau_{v}$ = 0.65, effectively capturing whitish or grayish smoke regions that often occur under dense atmospheric scattering.

Using the derived quantitative descriptors ($L_{f}$, $R_{f}$, *fire*, *smoke*), captions are generated through a series of predefined templates that translate physical measurements into natural language expressions. Examples include:

“A {L_f} fire covers approximately {R_f}% of the area with {smoke_status} smoke.”

“infrared data indicate {L_f} burning intensity with {smoke_status} smoke emission.”

To enhance linguistic diversity and reduce template bias, each caption is then expanded using large language model augmentation. An instruction-tuned model (e.g., GPT-4 or Mistral-Instruct) paraphrases each base caption into several semantically equivalent forms while preserving its physical meaning. For example, the base caption “A moderate fire covers around 30% of the area with light smoke” may be rewritten as:

“About one-third of the region is burning with moderate intensity and thin smoke.”

“A medium-scale fire affects roughly 30% of the area, producing light gray smoke.”

This hybrid annotation pipeline ensures that every caption in the dataset is both physically grounded and linguistically diverse. Across the complete FireMM-Instruct dataset, 83,000 caption–image pairs are generated, each providing a semantically faithful textual description aligned with the optical–infrared evidence of remote sensing forest fire scenes.

**Figure 4:**
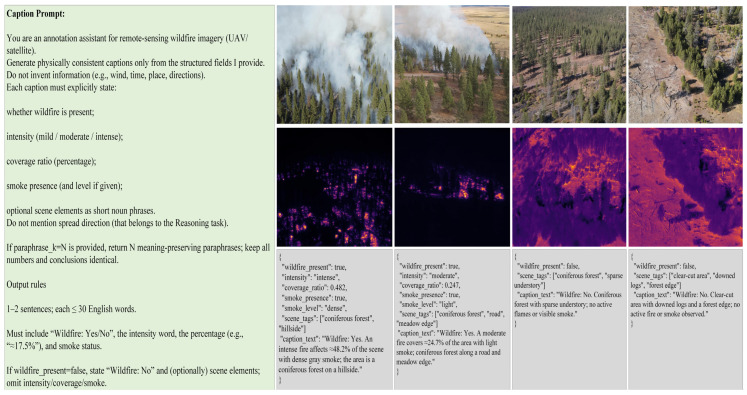
Caption prompt and examples in our FireMM-Instruct.

As shown in Figure 4, the instruction yields concise, physically grounded captions for both fire and no-fire scenes, while keeping numeric values unchanged (optionally with paraphrase_k for safe variants).

Reasoning labels in FireMM-Instruct are produced by a template-driven pipeline that infers a fire-spread direction from three visual cues—(1) the infrared source distribution in the infrared map, (2) smoke orientation and dispersion in the optical image, and (3) terrain/contextual cues visible in the scene—and then fills these in human-readable templates which are finally augmented by an LLM for linguistic variety. Concretely, we first compute an infrared field descriptor from the infrared channel T(x,y). Let P={(x,y)|M(x,y)=1} denote the set of fire pixels; we estimate the heat-weighted centroid:

(22) $$c_{T}=\frac{1}{\sum_{(x,y)\epsilon P}T(x,y)}\sum_{(x,y)\epsilon P}T(x,y){\left[x,y\right]}^{T}$$

and the infrared gradient vector field ∇T(x,y). A compact infrared direction vector is obtained by aggregating local gradients weighted by temperature:

(23) $$v_{T}=\sum_{(x,y)\epsilon P}T(x,y)\nabla T(x,y)$$

which points toward the direction of increasing infrared intensity and therefore suggests the likely expansion trend driven by active heating. Next, smoke orientation $v_{s}$ is estimated from the smoke mask S(x,y) obtained by the smoke detecto. We compute the principal axis of the smoke mask via PCA on the coordinates of smoke pixels:

(24) $$v_{s}=principal\;eiugenvector(Cov(\{(x,y)|S(x,y)\;=\;1\}))$$

where the eigenvector direction is disambiguated by the likely upwind/downwind sense using relative displacement between the infrared centroid $c_{T}$ and the smoke mask centroid. For terrain/context cues, when salient linear slope indicators or ridge/valley patterns are visually detectable, we extract a context vector $v_{G}$ from edge orientation statistics or image-derived hillshade proxies; when terrain cues are absent or unreliable, we set $v_{G}=0$.

These three vectors are fused into a single predicted spread vector using confidence weights ($W_{T}$, $W_{S}$, $W_{G}$) that reflect the reliability of each cue (e.g., infrared cue weight is high when $N_{f}$ is large; smoke cue weight increases with contiguous smoke extent). The fused vector is:

(25) $$v=\frac{W_{T}V_{T}+W_{S}V_{S}+W_{G}V_{G}}{\left\|W_{T}V_{T}+W_{S}V_{S}+W_{G}V_{G}+\varepsilon \right\|},\;\varepsilon ↓0\;$$

We convert v to an angular bearing $\theta =atan2(v_{x},v_{y})\;$ and discretize it into semantic directions (e.g., N, NE, E, SE, S, SW, W, NW) using fixed angular bins, producing a label such as “eastward” or “north-eastward.” A complementary confidence score c ∈ [0, 1] is computed from the normalized magnitude of the fused vector and the agreement among cues (high agreement → higher c), which is used to qualify the template (e.g., “likely to spread eastward” vs. “may spread eastward”).

The textual templates are designed to reflect both the predicted direction and the supporting visual evidence, for example: “The fire is likely to spread {DIRECTION} based on concentrated infrared gradients and smoke drift,” or “infrared hotspots and smoke plume orientation suggest a probable spread toward {DIRECTION}; confidence: {CONFIDENCE}.” Each filled template is then paraphrased by GPT-4 under constrained prompts that preserve the original inferred direction and the key supporting evidence, producing multiple semantically equivalent reasoning statements (e.g., “Given the high infrared gradient and smoke plume pointing east, the fire is expected to advance eastward.”/“Heat concentration and plume drift indicate an eastward spread is probable.”).

We emphasize that this PCA-based direction is defined in the image plane and serves only as a coarse supervisory signal for spread-direction inference, rather than as an absolute geo-referenced wind estimate for operational use.

**Figure 5:**
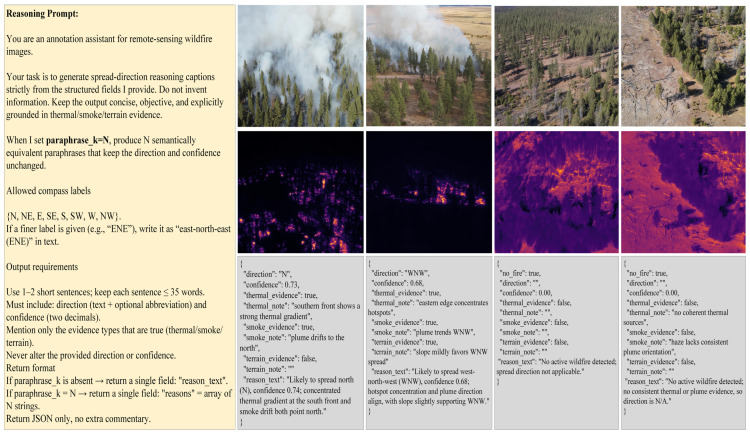
Reasoning prompt and examples in our FireMM-Instruct.

As shown in Figure 5, we verbalize pre-computed direction, confidence, and cue flags into short rationales using a constrained prompt; no-fire scenes return N/A with an explicit reason, and paraphrase_k provides controlled linguistic variants without altering labels. This hybrid, cue-based + LLM augmentation approach yields reasoning annotations that are grounded in image evidence, explicit about the visual basis for the inference, and sufficiently linguistically diverse to train FireMM-IR to perform independent, instruction-driven prediction tasks.

Given the safety-critical nature of wildfire monitoring, fully automated annotation is not yet reliable. We therefore rely on human annotators, guided by a detailed protocol, to draw pixel-wise masks and write instruction-style captions and reasoning descriptions. A subset of samples is further reviewed by remote-sensing and fire-management experts to verify semantic correctness and reduce systematic bias. However, this process also has limitations: ambiguous fire boundaries under dense smoke, varying expertise among annotators, and time constraints can introduce label noise and subjective interpretations, especially for high-level reasoning statements.

### 4.3. Dataset Statistics and Analysis

As shown in Table 1 and Table 2, most existing fire datasets are small, RGB-only, and single-task (e.g., AIDER/FireNet/FireDet for classification; UAVs-FFDB adds limited segmentation), while UAV aerial collections in the FLAME series either lack strict pairing (FLAME) or radiometry (FLAME-2), with FLAME-3 being the only radiometric source but much smaller. In contrast, FireMM-Instruct contributes 83k paired RGB–IR samples with radiometric IR, and uniquely supports captioning and reasoning in addition to segmentation/classification. We also provide instruction-aligned text via 12 base templates augmented by LLM paraphrasing, yielding linguistically diverse yet physically grounded supervision. Overall, FireMM-Instruct combines scale, pairing, radiometry, and instructional labels in a single corpus, offering substantially richer supervision than prior datasets and enabling holistic, multi-task wildfire understanding.

Table 1 provides a comprehensive comparison of publicly available forest fire datasets and the proposed FireMM-Instruct dataset. Early datasets such as AIDER, FireNet, and FireDet were primarily web-sourced and consisted solely of RGB images without radiometric calibration, limiting their use to basic classification tasks. With the introduction of UAV-based data acquisition, the FLAME series greatly improved spatial resolution and scene diversity, and gradually incorporated infrared modalities and paired imagery. However, except for FLAME 3, most datasets still lacked radiometric information, making them unsuitable for accurate temperature modeling or multimodal semantic reasoning. In contrast, the proposed FireMM-Instruct (2025) dataset unifies and extends the FLAME series, providing precisely aligned paired RGB–IR imagery with full radiometric data.

**Figure 6:**
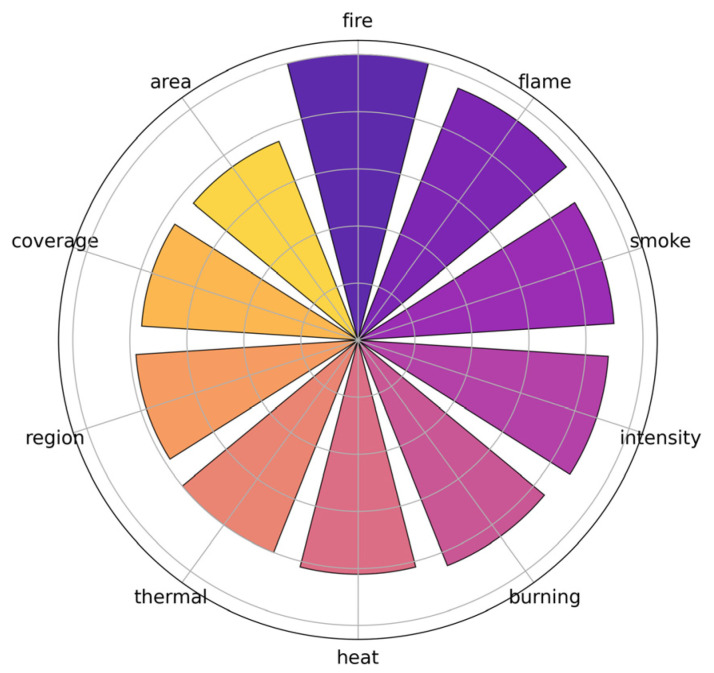
Display of some common keywords in the proposed FireMM-Instruct dataset.

Moreover, it includes multi-level annotations that support classification, captioning, reasoning, and segmentation tasks—offering a comprehensive foundation for training and evaluating multimodal large language models in remote sensing-based forest fire monitoring. Figure 6 illustrates the distribution of key descriptive terms in FireMM-Instruct, showing balanced coverage of visual and physical fire attributes such as smoke, flame, and infrared intensity. Although FireMM-Instruct covers a range of geographic locations, vegetation types, and atmospheric conditions, it does not exhaustively span all possible operational scenarios. Certain biomes, fuel complexes, and extreme weather situations remain under-represented. This means that model performance may degrade when extrapolating to regions with very different vegetation structure, topography, or sensor platforms from those seen in training. We discuss these limitations and the need for broader geographic and environmental coverage in the Discussion and Conclusions.

Table 2 summarizes the overall composition of the FireMM-Instruct dataset. The dataset contains a total of 83,000 paired RGB–IR samples, among which 54,000 correspond to fire scenes and 29,000 to non-fire backgrounds. The training and testing sets follow an approximate 4:1 ratio, ensuring a balanced representation of both fire and non-fire instances. To enable multimodal instruction tuning, each image pair is associated with caption and reasoning templates, including 12 manually designed base prompts that are further expanded through large language model (LLM) paraphrasing. This structure allows the dataset to support diverse multimodal learning tasks such as scene description, hazard reasoning, and visual–textual understanding in remote sensing-based fire monitoring.

## 5. Experiments and Discussion

### 5.1. Implementation Details

All experiments were conducted on the proposed FireMM-Instruct dataset using a multi-modal large-scale model, referred to as FireMM-IR, which integrates optical–infrared perception and instruction-aligned language reasoning within a unified framework.

FireMM-IR is implemented in PyTorch 2.3 and trained on 2 × NVIDIA A100 GPUs (80 GB) under a distributed data-parallel setup. Each RGB–IR pair is resized to 512 × 512 pixels, with RGB channels normalized by global z-score and infrared channels normalized using the radiometric scaling. The visual encoder adopts a dual-branch ViT-B/16 backbone, separately processing RGB and IR inputs, followed by a cross-modal fusion transformer that aligns multi-sensor features through a temperature-weighted gating mechanism. The language decoder is initialized from LLaVA-1.5 and further tuned for instruction-following on the captioning and reasoning tasks.

We use the AdamW optimizer with an initial learning rate of 2 × 10^−4^, cosine decay schedule, and weight decay = 0.05. Each mini-batch contains 64 image pairs, and training converges after 25 epochs (10 + 15).

The optimization uses a two-stage schedule. Stage I adopts task weights $\left(\lambda_{1},\lambda_{2},\epsilon )=(1.0,0.8,0.05\right)$, and we set $\left(\lambda_{1}^{\prime },\lambda_{2}^{\prime },\lambda_{3}^{\prime })=(0.5,0.5,2.0\right)$.

A compact Class-Aware Memory (CAM) module stores 128 prototype features (dimension = 512) to enhance cross-scene consistency.

We evaluate FireMM-IR under three complementary tasks—captioning, reasoning, and segmentation—to assess both linguistic and perceptual performance.

- Captioning: evaluated using BLEU-4, METEOR, ROUGE-L, and CIDEr to measure fluency, semantic relevance, and alignment with physical descriptors.
- Reasoning: quantified by Directional Accuracy (%)—the agreement between predicted and reference spread directions—and Factual Consistency (%), computed using a GPT-based textual comparison of physical evidence (infrared or smoke cues).
- Segmentation: assessed using mean Intersection-over-Union (mIoU).

All metrics are averaged over the held-out test split (16,600 pairs, 20%). Statistical significance is confirmed by repeating each experiment three times with different random seeds and reporting the mean. It is important to note that our directional inference head operates on a single RGB–IR frame and relies on apparent temperature gradients and spatial fire patterns. As a result, the estimated fire-spread direction can become uncertain in scenes where the thermal signal is weak or nearly uniform, in heavily smoke-obscured regions where the IR measurements are attenuated, or in complex situations where multiple fire fronts overlap or are strongly influenced by local wind and topography. The current design does not explicitly model temporal dynamics or multi-frame evolution, and should therefore be interpreted as a local, instantaneous estimate rather than a full multi-temporal propagation prediction.

### 5.2. Compare with Existing Models

We conduct three comparative studies on FireMM-Instruct under a unified evaluation: (1) pixel-level segmentation, contrasting four conventional semantic segmentation baselines (U-Net, DeepLabv3+, SegFormer-B3, Mask2Former; all RGB-only) and two LLM/promptable pipelines (LLaVA-1.5 + SAM2, GeoPix-style referring segmentation; RGB-only) against FireMM-IR (RGB+IR) with mIoU; (2) captioning, benchmarking five representative MLLMs (MiniGPT4v2, LLaVA-1.5, Mini-Gemini, GeoChat, GeoPix; RGB-only) versus FireMM-IR (RGB+IR) using BLEU-4, METEOR, ROUGE-L, CIDEr; and (3) reasoning (spread direction) on the same five MLLMs (RGB-only) versus FireMM-IR (RGB + IR) with Directional Accuracy and Factual Consistency.

We compare four conventional semantic segmentation baselines—U-Net (ResNet34), DeepLabv3+ (R101), SegFormer-B3, and Mask2Former (R50)—all trained with RGB only, against two LLM/promptable pipelines—LLaVA-1.5 + SAM2 (prompted mask decoding) and a GeoPix-style referring segmentation variant—also RGB only. Our method FireMM-IR uses RGB + IR fusion and the two-stage optimization. All models are trained at 512 × 512 with the same splits and evaluated by mIoU. Most existing operational and research systems for wildfire detection and mapping still rely on RGB or multispectral imagery without co-registered thermal channels, due to cost and payload constraints. Therefore, we adopt strong RGB-only segmentation networks (e.g., U-Net, DeepLabv3+, SegFormer, Mask2Former) as baselines, which reflect the current practice when infrared sensing is not available. Comparing FireMM-IR against these widely used RGB-only baselines highlights the additional value brought by the infrared modality and our dedicated fusion design.

**Figure 7:**
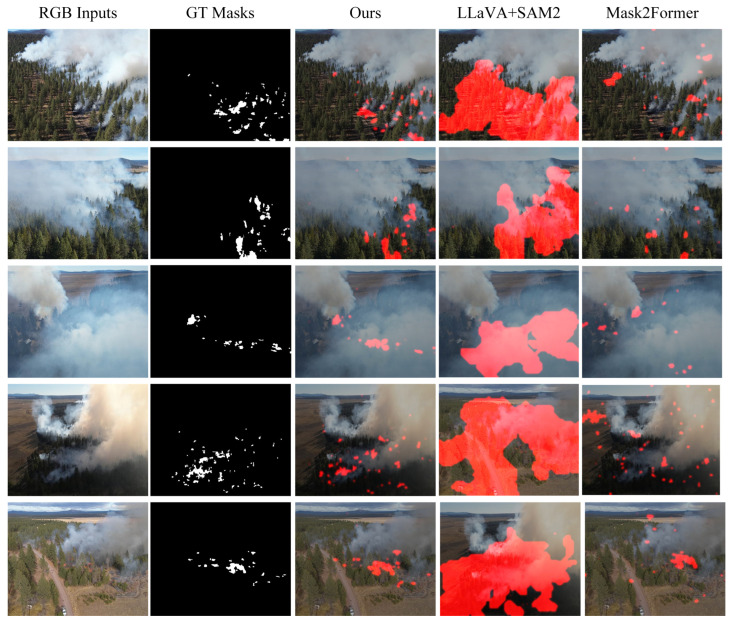
Qualitative comparison of active-flame segmentation on remote sensing images.

As shown in Table 3, FireMM-IR achieves the best mIoU, confirming the benefit of infrared–optical fusion for smoke-occluded and small-ignition regions. To complement the quantitative results, we visualize representative remote sensing fire scenes in Figure 7. Across complex scenarios such as dense smoke, small scattered fires, and cluttered forest backgrounds, FireMM-IR generates sharper and more localized masks that align well with the ground truth, while LLaVA+SAM2 tends to over-segment smoke regions and Mask2Former often fails to capture fine-scale hotspots. The improvements are particularly evident near smoke–canopy boundaries and in low-contrast conditions, underscoring the advantages of infrared–optical fusion and the proposed memory-enhanced reasoning mechanism for precise and physically consistent fire localization.

For Caption and reasoning benchmark, we benchmarked our FireMM-IR against five representative MLLMs: two general-purpose chat-vision models (MiniGPT4v2 [40], LLaVA-1.5 [39]), a lightweight general model (Mini-Gemini), and two remote-sensing–oriented models (GeoChat [2], GeoPix [27]). We evaluate instruction-driven caption quality on the FireMM-Instruct test split using BLEU-4, METEOR, ROUGE-L, and CIDEr [27], which jointly capture fluency, semantic relevance, and alignment with physical descriptors inferred from RGB–IR evidence. All competing MLLMs are fine-tuned on the same training split with RGB inputs; FireMM-IR (ours) uses RGB+IR and the two-stage optimization.

As shown in Table 4, FireMM-IR achieves the best overall captioning performance—improving ROUGE-L and CIDEr over RGB-only MLLMs—indicating that infrared–optical fusion plus staged optimization yields captions that better reflect physically grounded scene attributes. We evaluate instruction-driven spread-direction reasoning on the test split using Directional Accuracy (%)—agreement with the 8-way reference label—and Factual Consistency (%), computed by a GPT-based verifier that checks whether generated rationales align with infrared/smoke evidence. Baselines are fine-tuned with RGB only; FireMM-IR (ours) uses RGB+IR and the two-stage optimization.

As shown in Table 5, FireMM-IR achieves the best directional accuracy and factual consistency, indicating that infrared–optical fusion provides stronger physical grounding for spread-direction reasoning.

**Figure 8:**
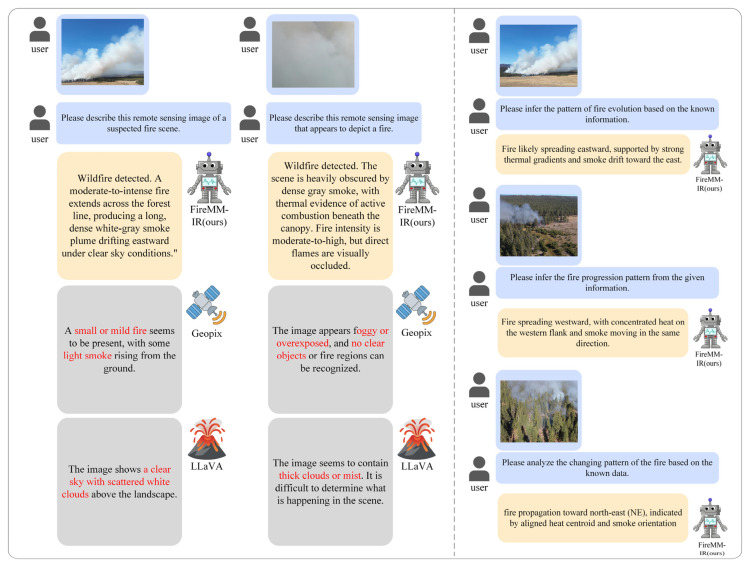
Qualitative results for captioning and reasoning on remote sensing fire scenes.

Figure 8 illustrates FireMM-IR’s language outputs on representative remote sensing images. Our model generates physically grounded captions that jointly reference fire intensity, areal extent, and smoke behavior, even under heavy occlusion. For reasoning prompts, FireMM-IR provides explicit, directionally consistent inferences (e.g., eastward, westward, NE) accompanied by evidence tied to infrared gradients and smoke drift, while baselines tend to produce generic weather comments or miss fire-specific cues. These results highlight the benefit of infrared–optical fusion and our memory-enhanced instruction head for accurate, evidence-based language understanding of wildfire scenes.

We also qualitatively examine how FireMM-IR extrapolates to new scenarios that differ from the training distribution, such as scenes with different vegetation patterns, terrain slopes, or sensor viewpoints. In many cases, the model is able to correctly highlight active fire fronts and generate coherent descriptions even when the background context (e.g., valley vs. ridge, sparse vs. dense forest) was under-represented in FireMM-Instruct. However, we observe occasional failures in rare configurations, such as highly reflective surfaces near the fire line or unusual smoke–cloud interactions, where the model may either overestimate or underestimate the affected area. These observations are consistent with our discussion on dataset coverage and underline the need for broader geographic and environmental diversity.

Across all three tasks, FireMM-IR is consistently superior to RGB-only competitors. In segmentation, it delivers the highest mIoU, indicating that infrared–optical fusion materially improves boundary localization under smoke and for small ignition regions. In captioning, FireMM-IR attains the best metrics, evidencing stronger physical grounding of language in infrared cues. For reasoning, it achieves the top Directional Accuracy and Factual Consistency, showing that radiometric information—combined with instruction-aligned training—enhances both the correctness of spread predictions and the alignment of rationales with observable evidence. Overall, the robustness experiments indicate that FireMM-IR degrades gracefully under adverse conditions. Dense smoke primarily reduces the effective thermal contrast and obscures visual cues, leading to softer fire boundaries, but the model still maintains a margin over RGB-only baselines as long as some residual hotspots remain visible. Artificial radiometric noise mainly affects small isolated regions and is partly mitigated by the temperature gating and spatial aggregation in CAM. Decreasing resolution gradually blurs fine-scale fronts and narrow fire lines, which is expected for any pixel-wise method, while partial RGB–IR misalignment mostly harms boundary accuracy where parallax is strongest. These results suggest that the proposed fusion strategy can generalize to moderately degraded conditions, but extremely dense smoke, severe mis-registration, or very low resolution will still challenge the model and should be treated with caution in operational use.

### 5.3. Ablation Studies

Beyond comparisons with existing methods, we conduct a suite of ablations to isolate the contribution of FireMM-IR. Unless otherwise stated, all ablations follow the same setting as mentioned in implementation details.

We first ablate the sensing modality and fusion strategy to quantify the value of infrared cues and the effectiveness of our temperature-aware gating. We compare RGB-only, IR-only, early concatenation, late summation, a variant without gating (fixed averaging), and the full model.

The results in Table 6 show that IR cues are beneficial but naïve fusion underexploits them: learned temperature-aware gating delivers the best performance, outperforming late fusion by +2.1 mIoU and RGB-only by +6.1 mIoU, which underscores the importance of adaptive cross-modal weighting for delineating smoke-occluded boundaries and small ignition regions.

We next examine the training schedule and loss weighting. Four variants are compared: a single-stage run using the Stage-II weights, removing the Stage-I weak pixel supervision (ε = 0.05), keeping Stage-I weights throughout (no Stage-II up-weight), and our two-stage scheme. Caption quality is co-reported to reflect language–vision alignment.

As shown in Table 7, the two-stage regime consistently improves spatial accuracy and caption grounding (+2.2 mIoU and +2.5 CIDEr over single-stage), confirming that early weak supervision stabilizes alignment while the later segmentation emphasis sharpens boundaries.

We then ablate instruction conditioning, reporting multi-instance prompt–mask assignment in addition to mIoU. Variants include removing instruction tokens, a promptable LLaVA-1.5 + SAM2 pipeline (RGB), an instruction-only variant without memory, and our instruction + CAM model. The results are shown in Table 8.

Instruction tokens enhance spatial disambiguation relative to no-instruction and promptable pipelines; adding CAM yields a further +1.2 mIoU and +2.5% assignment accuracy, indicating more stable multi-referring segmentation.

To quantify the effect of the memory design, we vary the number of prototypes K and the learning mode. A moderate, learnable memory is most effective.

As shown in Table 9, performance peaks at K = 128 with end-to-end updates; smaller or larger K slightly underperforms, and freezing prototypes reduces both segmentation and directional accuracy.

We also assess radiometry and pre-processing choices, contrasting true radiometric IR against pseudo-infrared normalization, per-sensor versus global normalization, and alignment robustness.

Radiometric calibration and per-sensor normalization offer measurable benefits, and performance degrades gracefully under controlled mis-registration, emphasizing the importance of accurate co-registration.

Finally, we study how the degree of language augmentation affects caption quality. For each image, a base template is instantiated from physical descriptors. Then an instruction-tuned LLM generates k paraphrases that preserve these descriptors under constrained prompts. We denote this as LLM-augmented (k×), meaning 1 base caption + k paraphrases per image; during training we sample uniformly from the caption pool (one caption per image per epoch). To reduce redundancy, near-duplicate paraphrases are filtered by semantic similarity and length constraints.

According to the results in Table 10, moderate augmentation (~3×) maximizes language metrics; heavier paraphrasing brings marginal returns, likely due to increased redundancy.

To further verify that the above observations are not limited to our self-constructed FireMM-Instruct benchmark, we additionally replicate the same ablation settings on the public FLAME-2 dataset using its official split. The results are summarized in Table 11. We observe a consistent trend: single-modality variants RGB-only and IR-only underperform multi-modal fusion, achieving 69.0 and 66.8 mIoU, respectively, while simple early and late fusion improve performance to 72.4 and 73.3 mIoU. Removing the temperature-aware gating module further increases the score to 74.0 mIoU, but it still lags behind the full FireMM-IR, which reaches 74.9 mIoU.

In other words, the full model improves over the best single-modality variant (RGB-only) by 5.9 mIoU and over simple early/late fusion by 2.5/1.6 mIoU, and the temperature-aware gating itself contributes an additional 0.9 mIoU. These findings indicate that the proposed fusion strategy and temperature-aware gating generalize well to a public RGB--IR wildfire dataset, rather than only bringing gains on FireMM-Instruct.

### 5.4. Discussion

From a sensing perspective, thermal infrared imagery has inherent limitations: sensor noise, calibration drift, and saturation can occur, especially near very intense fire cores or when the dynamic range is limited. Smoke and atmospheric effects may attenuate or scatter the thermal signal, causing underestimation of fire intensity or ambiguous hotspots. Moreover, the current FireMM-IR design relies on single-image inference and does not explicitly model temporal dynamics, which limits its ability to capture rapid propagation, spotting events, or multi-day evolution.

In addition, the proposed architecture, which combines dual encoders, temperature-aware fusion, CAM, and an LLM head, is more computationally demanding than lightweight CNN-based baselines. While this is acceptable for offline analysis and near-real-time applications on modern GPUs, it may be challenging for deployment on very constrained edge platforms without further model compression or distillation. These aspects should be taken into account when designing operational systems.

Another important aspect is uncertainty. The estimated fire-spread direction is derived from spatial patterns and temperature gradients within a single RGB–IR frame, and may be unreliable when the thermal signal is weak, heavily occluded by smoke, or dominated by complex wind–topography interactions. Similarly, segmentation masks are more uncertain near boundaries, in partially saturated regions, or in areas where the distinction between active fire and residual heating is ambiguous. In this work, we do not yet provide explicit uncertainty maps or calibrated confidence scores, but the qualitative behavior of the model suggests that incorporating uncertainty estimation (e.g., through Monte Carlo sampling, ensembles, or auxiliary confidence heads) is a promising direction for future research.

## 6. Conclusions

Motivated by the need to move beyond standalone detection/segmentation toward holistic, physically grounded wildfire understanding, we introduced FireMM-IR and FireMM-Instruct. FireMM-IR couples a dual-branch ViT-B/16 for RGB/IR with temperature-aware fusion, a LLaVA-initialized multi-task instruction head, and a compact CAM module for cross-scene consistency, enabling instruction-aligned captioning/reasoning alongside pixel-level perception. FireMM-Instruct contributes 83k geometrically aligned RGB–IR pairs with radiometric IR, instance masks, and instruction-aligned text (12 base templates with LLM paraphrasing), providing the scale and supervision breadth missing in prior corpora.

Despite these promising results, our study has several limitations. FireMM-Instruct, while large and diverse, does not cover all geographic regions, vegetation types, or sensor configurations encountered in real operations, and the current model does not explicitly exploit multi-temporal information. The reliance on thermal infrared imagery also makes the system sensitive to sensor calibration, saturation, and smoke attenuation in extreme conditions, and the computational complexity of the architecture may hinder deployment on very resource-limited platforms. Future work will investigate multi-temporal extensions of FireMM-IR, broader and more heterogeneous datasets, explicit uncertainty estimation, and lighter-weight variants tailored for edge devices.

On this benchmark, FireMM-IR attains 78.2 mIoU for segmentation, strong captioning quality (BLEU-4 15.9/METEOR 24.6/ROUGE-L 38.7/CIDEr 36.5), and robust reasoning (78.4% directional accuracy, 82.7% factual consistency). Ablations confirm the sources of gain: RGB+IR with temperature-aware fusion > naïve/uni-modal settings; the two-stage schedule improves spatial accuracy and language grounding; instruction conditioning + CAM strengthens multi-instance binding; and fusing infrared/smoke/terrain cues yields the most reliable spread-direction predictions.

Overall, the synergy of radiometric sensing, instruction alignment, and multi-task supervision makes FireMM-IR/FireMM-Instruct a practical, reproducible foundation for remote-sensing wildfire monitoring. Future work will extend the corpus with temporal (pre/post-burn) sequences and explore lightweight deployments for on-board UAV inference.

## Figures and Tables

**Table 1:** Comparison of existing forest fire datasets and the proposed FireMM-Instruct dataset in terms of collection platform, imaging modality, radiometric availability, and application diversity.

Dataset/Year	Collection, Perspective	Image Type	Pre/Post Burn Data?	Radiometric Data?	Applications	Image Count
AIDER [28], 2020	Web	RGB	No	No	Classification	1000
FireNet [29], 2019	Web	RGB	No	No	Classification	1900
FireDet [30], 2020	web	RGB	No	No	Classification	3225
FireNetv2 [31], 2023	web	RGB	No	No	Object detection	502
UAVs-FFDB [24], 2024	UAV	RGB	Yes	No	Classification; Modeling; Segmentation	1635
FLAME [32], 2020	UAV, Aerial	RGB/infrared (not pairs)	No	No	Classification; Segmentation	47,992
FLAME 2 [33], 2022	UAV, Aerial	RGB/infrared Pairs	Yes	No	Classification	53,451
FLAME 3 [34], 2024	UAV, Aerial	Dual RGB/IR + Radiometric infrared TIFF	Yes	Yes	Classification; Modeling; Segmentation	13,997
FireMM-Instruct (ours), 2025	UAV, Aerial (unified from FLAME series)	Paired RGB–IR Radiometric	No	Yes	Classification; Captioning; Reasoning; Segmentation;	83,000 pairs

**Table 2:** Composition and annotation details of the FireMM-Instruct dataset, including image pair distribution and caption–reasoning template design.

Split	Image Pairs	Fire Images	Non-Fire Images	Caption & Reasoning Templates
Train set	66,400	43,200	23,200	12 base templates + LLM paraphrases
Test set	16,600	10,800	5800	
Total set	83,000	54,000	29,000	

**Table 3:** Pixel-level segmentation on FireMM-Instruct. The symbol indicates that higher values are better.

Group	Method	Input	mIoU ↑
Conventional	U-Net (ResNet34) [35]	RGB	66.8
	DeepLabv3+ (R101) [36]	RGB	69.6
	SegFormer-B3 [37]	RGB	70.4
	Mask2Former (R50) [38]	RGB	71.2
LLM/Promptable	LLaVA-1.5 + SAM2 (prompt) [39]	RGB	22.7
	GeoPix-style referring seg [27]	RGB	43.9
Ours	FireMM-IR	RGB + IR	78.2

**Table 4:** Captioning performance on FireMM-Instruct. The symbol indicates that higher values are better.

Method	BLEU-4 ↑	METEOR ↑	ROUGE-L ↑	CIDEr ↑
MiniGPT4v2 [40]	8.7	17.1	30.8	21.4
LLaVA-1.5 [39]	14.7	21.9	36.9	33.9
Mini-Gemini [41]	14.3	21.5	36.8	33.5
GeoChat [2]	13.8	21.1	35.2	28.2
GeoPix [27]	14.0	23.4	36.3	31.3
FireMM-IR (ours)	15.9	24.6	38.7	36.5

**Table 5:** Reasoning performance on FireMM-Instruct. The symbol indicates that higher values are better.

Method	Directional Accuracy (%) ↑	Factual Consistency (%) ↑
MiniGPT4v2 [40]	61.3	72.6
LLaVA-1.5 [39]	66.8	75.2
Mini-Gemini [41]	66.1	74.1
GeoChat [2]	68.7	76.4
GeoPix [27]	71.5	77.3
FireMM-IR (ours)	78.4	82.7

**Table 6:** Ablation on modality and fusion design. The symbol indicates that higher values are better.

Setting	mIoU ↑
RGB-only	72.1
IR-only	70.4
Early fusion (concat)	75.0
Late fusion (sum)	76.1
w/o temperature gating	76.8
Ours (full)	78.2

**Table 7:** Ablation of optimization strategy on FireMM-Instruct. The symbol indicates that higher values are better.

Setting	mIoU ↑	CIDEr ↑
Single-stage	76.0	34.0
w/o Stage-I ε	76.5	34.8
w/o Stage-II up-weight	75.4	34.1
Two-stage (ours)	78.2	36.5

**Table 8:** Ablation on instruction grounding and CAM. The symbol indicates that higher values are better.

Setting	Input	mIoU ↑	Prompt→Mask Match Acc. (%) ↑
No instruction tokens	RGB+IR	74.3	88.5
LLaVA-1.5 + SAM2 [39]	RGB	73.9	90.7
Instruction-only (no CAM)	RGB+IR	77.0	92.1
Instruction + CAM (ours)	RGB+IR	78.2	94.6

**Table 9:** Ablation on Class-Aware Memory (CAM): effect of memory size and learnability. The symbol indicates that higher values are better.

Setting	mIoU ↑	Directional Acc. (%) ↑
w/o CAM	77.0	76.1
CAM (K = 64)	77.6	77.4
CAM (K = 128, learnable)	78.2	78.4
CAM (K = 256, learnable)	78.1	78.3
CAM (K = 128, frozen)	77.3	77.0

**Table 10:** Impact of template augmentation on captioning: effect of LLM paraphrase scale on FireMM-Instruct. The symbol indicates that higher values are better.

Setting	BLEU-4 ↑	METEOR ↑	ROUGE-L ↑	CIDEr ↑
Templates-only	15.1	23.5	37.2	34.2
LLM-augmented (1×)	15.6	24.1	38.0	35.3
LLM-augmented (3×, ours)	15.9	24.6	38.7	36.5
LLM-augmented (5×)	15.8	24.6	38.6	36.4

**Table 11:** Ablation on the public FLAME-2 test set. The symbol indicates that higher values are better.

Setting	mIoU ↑
RGB-Only	69.0
IR-Only	66.8
Early fusion (concat)	72.4
w/o temperature gating	74.0
Ours (full)	74.9

## Data Availability

The original contributions presented in this study are included in the article. Further inquiries can be directed to the corresponding author.

## References

[1] Barmpoutis P., Papaioannou P., Dimitropoulos K., Grammalidis N. (2020). A review on early forest fire detection systems using optical remote sensing. Sensors.

[2] Carta F., Zidda C., Putzu M., Loru D., Anedda M., Giusto D. (2023). Advancements in forest fire prevention: A comprehensive survey. Sensors.

[3] Daryal U., Giri A., Karki S., Lepcha P., Alam S., Singh A.P. (2025). Early Warning Systems: Enhancing Fire Prediction and Response. Forest Fire and Climate Change: Insights into Science.

[4] Elvidge C.D., Baugh K.E., Ziskin D., Anderson S., Ghosh T. (2011). Estimation of Gas Flaring Volumes Using NASA MODIS Fire Detection Products.

[5] Escuin S., Navarro R., Fernández P. (2008). Fire severity assessment by using NBR (Normalized Burn Ratio) and NDVI (Normalized Difference Vegetation Index) derived from LANDSAT TM/ETM images. Int. J. Remote Sens..

[6] Frey R.A., Ackerman S.A., Holz R.E., Dutcher S., Griffith Z. (2020). The continuity MODIS-VIIRS cloud mask. Remote Sens..

[7] Töreyin B.U., Dedeoğlu Y., Güdükbay U., Çetin A.E. (2006). Computer vision based method for real-time fire and flame detection. Pattern Recognit. Lett..

[8] Han K., Wang Y., Chen H., Chen X., Guo J., Liu Z., Tang Y., Xiao A., Xu C., Xu Y. (2022). A survey on vision transformer. IEEE Trans. Pattern Anal. Mach. Intell..

[9] Chen H., Gao S., Xiang L., Cai C., Wang C. (2023). FIRE-DET: An efficient flame detection model. Nanjing Xinxi Gongcheng Daxue Xuebao.

[10] Hopkins B., O’Neill L., Afghah F., Razi A., Rowell E., Watts A., Fule P., Coen J. (2023). Flame 2: Fire detection and modeling: Aerial multi-spectral image dataset. IEEE DataPort.

[11] Hopkins B., ONeill L., Marinaccio M., Rowell E., Parsons R., Flanary S., Nazim I., Seielstad C., Afghah F. (2024). Flame 3 dataset: Unleashing the power of radiometric thermal uav imagery for wildfire management. arXiv.

[12] Hu Y., Yuan J., Wen C., Lu X., Liu Y., Li X. (2025). Rsgpt: A remote sensing vision language model and benchmark. ISPRS J. Photogramm. Remote Sens..

[13] Huang H., Zheng O., Wang D., Yin J., Wang Z., Ding S., Yin H., Xu C., Yang R., Zheng Q. (2023). ChatGPT for shaping the future of dentistry: The potential of multi-modal large language model. Int. J. Oral Sci..

[14] Jadon A., Omama M., Varshney A., Ansari M.S., Sharma R. (2019). FireNet: A specialized lightweight fire & smoke detection model for real-time IoT applications. arXiv.

[15] Kim J.T., Oh T.S., Lee D.H. (2003). Preparation and characteristics of nitrile rubber (NBR) nanocomposites based on organophilic layered clay. Polym. Int..

[16] Kuckreja K., Danish M.S., Naseer M., Das A., Khan S., Khan F.S. Geochat: Grounded large vision-language model for remote sensing. Proceedings of the IEEE/CVF Conference on Computer Vision and Pattern Recognition.

[17] Li Y., Zhang Y., Wang C., Zhong Z., Chen Y., Chu R., Liu S., Jia J. (2024). Mini-gemini: Mining the potential of multi-modality vision language models. arXiv.

[18] Li Z., Liu F., Yang W., Peng S., Zhou J. (2021). A survey of convolutional neural networks: Analysis, applications, and prospects. IEEE Trans. Neural Netw. Learn. Syst..

[19] Lin B., Ye Y., Zhu B., Cui J., Ning M., Jin P., Yuan L. Video-llava: Learning united visual representation by alignment before projection. Proceedings of the 2024 Conference on Empirical Methods in Natural Language Processing.

[20] Manakitsa N., Maraslidis G.S., Moysis L., Fragulis G.F. (2024). A review of machine learning and deep learning for object detection, semantic segmentation, and human action recognition in machine and robotic vision. Technologies.

[21] Moldamurat K., La Spada L., Zeeshan N., Bakyt M., Kuanysh A., bi Zhanibek K., Tilenbayev A. (2025). AI-Enhanced High-Speed Data Encryption System for Unmanned Aerial Vehicles in Fire Detection Applications. J. Robot. Control. (JRC).

[22] Mowla M.N., Asadi D., Tekeoglu K.N., Masum S., Rabie K. (2024). UAVs-FFDB: A high-resolution dataset for advancing forest fire detection and monitoring using unmanned aerial vehicles (UAVs). Data Brief.

[23] Muhammad K., Ahmad J., Baik S.W. (2018). Early fire detection using convolutional neural networks during surveillance for effective disaster management. Neurocomputing.

[24] Muhtar D., Li Z., Gu F., Zhang X., Xiao P. Lhrs-bot: Empowering remote sensing with vgi-enhanced large multimodal language model. Proceedings of the European Conference on Computer Vision.

[25] Ou R., Hu Y., Zhang F., Chen J., Liu Y. (2025). GeoPix: A multimodal large language model for pixel-level image understanding in remote sensing. IEEE Geosci. Remote Sens. Mag..

[26] Kim H.R., Ko B.C. (2025). Keyword-Conditioned Image Segmentation via the Cross-Attentive Alignment of Language and Vision Sensor Data. Sensors.

[27] Persello C., Wegner J.D., Hänsch R., Tuia D., Ghamisi P., Koeva M., Camps-Valls G. (2022). Deep learning and earth observation to support the sustainable development goals: Current approaches, open challenges, and future opportunities. IEEE Geosci. Remote Sens. Mag..

[28] Ronneberger O., Fischer P., Brox T. U-net: Convolutional networks for biomedical image segmentation. Proceedings of the International Conference on Medical Image Computing and Computer-Assisted Intervention.

[29] Shamsoshoara A., Afghah F., Razi A., Zheng L., Fulé P.Z., Blasch E. (2021). Aerial imagery pile burn detection using deep learning: The FLAME dataset. Comput. Netw..

[30] Shees A., Ansari M.S., Varshney A., Asghar M.N., Kanwal N. (2023). Firenet-v2: Improved lightweight fire detection model for real-time iot applications. Procedia Comput. Sci..

[31] Sivachandra K., Kumudham R. (2024). A review: Object detection and classification using side scan sonar images via deep learning techniques. Modern Approaches in Machine Learning and Cognitive Science: A Walkthrough.

[32] Tran A., Tran M., Marti E., Cothren J., Rainwater C., Eksioglu S., Le N. (2025). Land8Fire: A Complete Study on Wildfire Segmentation Through Comprehensive Review, Human-Annotated Multispectral Dataset, and Extensive Benchmarking. Remote Sens..

[33] Tu H., Cui C., Wang Z., Zhou Y., Zhao B., Han J., Zhou W., Yao H., Xie C. (2023). How many unicorns are in this image? A safety evaluation benchmark for vision llms. arXiv.

[34] Vermote E., Roger J.-C., Franch B., Skakun S. LaSRC (Land Surface Reflectance Code): Overview, application and validation using MODIS, VIIRS, LANDSAT and Sentinel 2 data’s. Proceedings of the IGARSS 2018—2018 IEEE International Geoscience and Remote Sensing Symposium.

[35] Wang Z., Shi D., Qiu C., Jin S., Li T., Shi Y., Liu Z., Qiao Z. (2024). Sequence matching for image-based uav-to-satellite geolocalization. IEEE Trans. Geosci. Remote Sens..

[36] Wu H., Liu Q., Liu X. (2019). A review on deep learning approaches to image classification and object segmentation. Comput. Mater. Contin.

[37] Xie E., Wang W., Yu Z., Anandkumar A., Alvarez J.M., Luo P. (2021). SegFormer: Simple and efficient design for semantic segmentation with transformers. Adv. Neural Inf. Process. Syst..

[38] Yurtkulu S.C., Şahin Y.H., Unal G. Semantic segmentation with extended DeepLabv3 architecture. Proceedings of the 2019 27th Signal Processing and Communications Applications Conference (SIU).

[39] Zhan Y., Xiong Z., Yuan Y. (2025). Skyeyegpt: Unifying remote sensing vision-language tasks via instruction tuning with large language model. ISPRS J. Photogramm. Remote Sens..

[40] Zhang G., Navasardyan S., Chen L., Zhao Y., Wei Y., Shi H. (2022). Mask matching transformer for few-shot segmentation. Adv. Neural Inf. Process. Syst..

[41] Zhang W., Cai M., Zhang T., Zhuang Y., Mao X. (2024). EarthGPT: A universal multimodal large language model for multisensor image comprehension in remote sensing domain. IEEE Trans. Geosci. Remote Sens..

